# Novel biomarkers to guide glucocorticoid replacement in primary adrenal insufficiency: a proof-of-concept study using copeptin-derived ratios

**DOI:** 10.1007/s40618-026-02874-8

**Published:** 2026-04-24

**Authors:** Chiara Bima, Lorenzo Campioni, Alessandro Maria Berton, Chiara Sola, Beatrice Bergoglio, Maria Chiara Di Carlo, Federico Ponzetto, Fabio Settanni, Filippo Ceccato, Mirko Parasiliti-Caprino, Roberta Giordano

**Affiliations:** 1 AOU Città Salute e Scienza di Torino, Torino, Italy; 2 Department of Medical Sciences, University of Turin, Turin, Italy; 3 Clinical Biochemistry Laboratory, City of Health and Science University Hospital, Turin, Italy; 4 Endocrinology Unit, Department of Medicine DIMED, University-Hospital of Padua, Padua, Italy; 5 University of Turin (Department of Biological and Clinical Sciences), Orbassano, Italy

**Keywords:** Primary adrenal insufficiency, Copeptin, Salivary cortisone, Salivary cortisol, Glucocorticoid replacement adequacy

## Abstract

**Background:**

Optimising glucocorticoid (GC) replacement in primary adrenal insufficiency (PAI) remains challenging, as clinical and biochemical markers do not reliably mirror tissue cortisol exposure.

**Objective:**

To evaluate salivary cortisone exposure and copeptin-derived ratios as candidate biomarkers of GC replacement adequacy in PAI.

**Design:**

Cross-sectional case–control study with 12-month follow-up including nineteen adults with autoimmune or idiopathic PAI on stable hydrocortisone (HC; immediate- or dual-release) plus fludrocortisone, and forty-three healthy controls.

**Methods:**

Six daytime saliva samples were collected for cortisol and cortisone quantification by LC–MS/MS. The area-under-the-curve (AUC) of cortisone was computed, and patients were classified as *GC excess* (AUC above the 90th percentile of controls; Group A) or *adequate replacement* (10th–90th percentile; Group B). Plasma ACTH, copeptin, renin, electrolytes and osmolarity were measured fasting and 120 min post-dose.

**Results:**

At baseline, 31.6% of patients showed GC excess, increasing to 36.8% at follow-up. Compared with Group B, Group A exhibited higher HbA1c (46.5 vs. 34.0 mmol/mol, *p* < 0.05) and diastolic blood pressure (84.5 ± 8.7 vs. 77.0 ± 9.6 mmHg, *p* < 0.05) with lower pulse pressure. ACTH and ACTH/copeptin ratios were markedly reduced in Group A both fasting (median 38.1 vs. 132.4) and post-dose (12.9 vs. 41.6), while renin/copeptin ratios declined at T1. ACTH/copeptin correlated inversely with cortisone AUC (*r* = − 0.59, *p* < 0.01).

**Conclusions:**

Salivary cortisone AUC, together with ACTH/copeptin and renin/copeptin ratios, identifies physiologically distinct GC exposure profiles linked to metabolic and haemodynamic alterations. These biomarkers may refine personalised GC replacement in PAI.

**Supplementary Information:**

The online version contains supplementary material available at 10.1007/s40618-026-02874-8.

## Introduction

Primary adrenal insufficiency (PAI), or Addison’s disease (AD), is a serious medical condition which, if untreated, has a high morbidity and mortality [1–3].

Standard management of PAI requires lifelong glucocorticoids (GC) and mineralocorticoid (MC) replacement therapy, with the therapeutic goal to restore cortisol levels, electrolyte and metabolic balance [4–6].

Conventional GC regimens using hydrocortisone (HC) or cortisone acetate (CA) fail to replicate the physiological circadian rhythm of cortisol secretion [7], and patients with PAI may experience periods of both under- and over-exposure to GC, with increased morbidity and mortality compared with the general population, mainly due to adverse cardiovascular and metabolic effects [8–10].

Recent studies have demonstrated that dual-release hydrocortisone (DR-HC) can more closely reproduce the physiological cortisol circadian profile [11–13], and it has been associated with improved blood pressure control, glucose and lipid metabolism, body weight, and health-related quality of life [11, 14–19].

Monitoring and management of GC replacement therapy remain challenging due to the lack of objective hormonal markers of adequacy [4, 5, 20]. Several methods have been proposed to assess treatment quality, yet none has achieved consensus for clinical use [21]. Among them, salivary cortisol evaluation has been considered as a practical tool for monitoring GC replacement, since it can be collected in outpatients and measurement by liquid chromatography coupled to mass spectrometry (LC–MS/MS) is not affected by CBG [13, 22, 23]. Moreover, assessment of salivary cortisone—a cortisol metabolite—has been introduced as an alternative marker, though results from the literature remain debated [24–27].

Measurement of plasma ACTH has not been considered a good tool as its normalization often leads to over-replacement [4]. Since ACTH synthesis and secretion are physiologically regulated by corticotropin-releasing hormone (CRH) and arginine vasopressin (AVP) [28] via negative feedback, these neurohormones may provide additional insight into GC replacement adequacy. Copeptin, the C-terminal portion of the precursor pre-provasopressin (CT-proAVP), is a reliable and stable surrogate marker of AVP secretion [29]. Several studies have highlighted its diagnostic and prognostic value in cardiovascular [30–32] and electrolyte disorders, including the differential diagnosis of hyponatraemia [33] and in polyuria–polydipsia syndromes [34–37], as well as in critical illness [38]. However, its potential role in adrenal insufficiency has not yet been defined.

Regarding MC replacement, direct renin concentration (DRC) or plasma renin activity (PRA) have been proposed as biochemical indicators, with PRA values in the upper reference range suggesting adequate replacement [39]. Nonetheless, the physiological complexity of PRA regulation limits its precision for dose titration [40].

Therefore, to date, GC treatment monitoring largely relies on clinical judgement, evaluating daily performance, subjective well-being, and the presence of signs or symptoms of GC over-replacement (weight gain, hypertension, hyperglycaemia, dyslipidaemia, osteoporosis) or under-replacement (fatigue, nausea, myalgia, joint stiffness, hypotension, hyperpigmentation, weight loss) [4–6, 20, 41]. Similarly, MC therapy management is mainly guided by clinical parameters—such as salt craving, oedema, and blood pressure—together with laboratory indices like serum sodium and potassium concentrations [4–6, 40]. Based on these considerations, the aim of the present study was to investigate novel biochemical markers of GC replacement adequacy in patients with PAI.

## Subjects and methods

### Design and study population

We designed a case control, cross-sectional study, conducted on adult patients affected by PAI in replacement therapy, referred to the Division of Endocrinology, Diabetes and Metabolism of the City of Health and Science University Hospital of Turin, enrolled between January 2021 and January 2022. Inclusion criteria were (a) age ≥ 18 years old; (b) diagnosis of PAI according to the Endocrine Society Guidelines [4] and the Italian Society of Endocrinology Position Statement [6]; (c) replacement therapy with conventional HC/DR- HC and fludrocortisone at stable dosage for at least 3 months.

Exclusion criteria were (a) age < 18 years old; (b) presence of severe cardio-, cerebrovascular, respiratory, hepatobiliary or pancreatic diseases, renal dysfunction, intestinal or gastric motility disorders and autoimmune systemic disease in steroid suppressive treatment.

The aetiology of adrenal insufficiency was assessed at diagnosis through measurement of anti 21-hydroxylase antibodies (21OHAbs), 17-hydroxyprogesterone, adrenal CT scan or very long chain fatty acids (VLCFAs) in males [4]. All women with primary ovarian insufficiency were under appropriate hormonal replacement therapy (HRT).

The study included two observation times: (a) T0: time of enrolment; (b) T1: after 12 months of follow up.

As a control group, we investigated a population of 43 healthy subjects, whose data were provided from the Endocrinology Unit, Department of Medicine (DIMED) of the University Hospital of Padua.

Healthy controls were all voluntary adults recruited among hospital employees and their family members; none of them were taking exogenous glucocorticoids or drugs that might interfere with the HPA axis; female volunteers were not taking oral or transdermal contraceptives and were investigated in the early follicular phase of the menstrual cycle. The control group was matched for age (44.74 ± 17.17, *p* = 0.795) and sex (19 males and 24 females, *p* = 0.788).

The study was performed in accordance with the guidelines in the Declaration of Helsinki and approved by the Ethics Committee of City of Health and Science University Hospital of Turin (N° 108444; 10/11/2020). Written informed consent was obtained from all enrolled subjects.

## Clinical investigation

Body weight, height, waist circumference, blood pressure and heart rate were measured using standard methods at time T0 and T1. Body mass index (BMI) was calculated (weight divided per height squared, kg/m^2^). Health related quality of life (HR QoL) was evaluated by a 30-item questionnaire (AddiQoL), purposely developed and validated in patients with AD in European subjects [41, 42].

## Biochemical evaluation

Both PAI patients and healthy controls performed a multiple daily saliva collection during normal routine day, for the assessment of salivary cortisol (Kendall’s compound F) and cortisone (Kendall’s compound E) profile.

Patients were advised to soak the absorbent cotton for 2–3 min, then the saliva sample was placed in a syringe and kept at + 4 °C. Samples were collected at least 30 min before eating or drinking, to avoid any source of food contamination; patients brushed their teeth at least 30 min before collecting their saliva. Smoking or eating liquorice was forbidden. The protocol was described in detail to the patients through a written form, to ensure a correct salivary sampling. PAI patients collected the samples during the week before their evaluation at time T0 and T1.

The times of collection were 0) on awakening/before taking replacement therapy; (1) 90 min after therapy; (2) 6 h after therapy/before lunch; (3) 8 h and 30 min after therapy; (4) 12 h after therapy/before dinner; (5) before sleeping. F0→F5 and E0→E5 indicate cortisol and cortisone measurements corresponding to the five salivary sampling time points. To estimate the individual exposure to glucocorticoid (GC) therapy, in both PAI patients and healthy controls, areas under the curve (AUCs) for salivary cortisol (AUC_F0→F5) and cortisone (AUC_E0→E5) were calculated using the trapezoid rule [43]. Classification based on the 10th–90th percentiles of control AUC_E0→E5 was adopted as an exploratory, physiology-anchored approach for this proof-of-concept study, rather than as a validated clinical cut-off.

At time T0 and T1, blood samples were collected from all patients fasting in the morning between 7.00 and 9.00 a.m. before and 120 min after GC and MC replacement therapy. Before taking replacement therapy, the following laboratory analytes were measured: copeptin, ACTH, renin, sodium (Na), potassium (K), creatinine, glucose, glycated hemoglobin (HbA1c), plasma osmolality (p-Osm), total cholesterol, HDL cholesterol, triglycerides. Copeptin, ACTH and renin were also measured 120 min after taking GC and MC replacement therapy. Urine samples were collected for the measurement of urine osmolality (u-Osm), spot urinary sodium (U-Na) and spot urinary potassium (U-K).

The 90-minute post-dose time point for salivary cortisol/cortisone was chosen to capture the expected peak following oral hydrocortisone, consistent with published salivary profiling studies [13, 23]. Plasma samples were collected before and 120 min after dosing in order to align with routine clinical sampling practices and to provide a standardized assessment of systemic cortisol and cortisone concentrations at a later post-dose time point and to assess systemic hormonal feedback (ACTH, copeptin, renin), which may occur later than the cortisol peak.

## Analytical methods

Salivary cortisol and cortisone (ng/ml) were determined on salivary samples collected by patients using a cotton-based sampling device called Salivette^®^ (SARSTEDT, Nümbrecht, Germany) through MassChrom^®^ Cortisol, Cortisone in Saliva LC MS/MS (Chromsystems Instruments & Chemicals GmbH, Gräfelfing, Germany). The quantification was performed on Nexera UHPLC system (Shimadzu, Kyoto, Japan) associated with a triple quadrupole mass spectrometry 4500MD (AB Sciex, Framingham, MA, USA). The assay sensitivity was 0.28 ng/ml for cortisol and 0.55 ng/ml for cortisone, intra and inter assay coefficients of variation were, respectively, below 5.1% and 8.8% for cortisol and below 4.9% and 8.8% for cortisone. To assess endogenous daily cortisol exposure, we calculated the Area Under the Curve (AUC) for salivary cortisol and cortisone levels according to the trapezoidal formula proposed by Pruessner [43]. Plasma ACTH levels (ng/L) were measured by chemiluminescent immunometric assay (CLIA) using LIAISON Analyzer (DiaSorin, Saluggia, Italy), with an analytical sensibility of 1.6 ng/l, and intra- and inter-assay coefficients of variation below 4.9 and 8.8% rspectively. Plasma copeptin (pmol/L) concentrations were determined with the B.R.A.H.M.S. KRYPTOR compact PLUS^®^ (Thermo Fisher Scientific, Hennigsdorf, Germany) automated method using the TRACE (Time Resolved Amplified Cryptate Emission) technique, an immunofluorescent analysis, which measures the delayed fluorescent signal transferred from donor molecules when bound in an immunocomplex. The detection limit of the assay was 0.9 pmol/L, while intra- and inter-assay coefficients of variation were below 7% and below 12%, respectively.

The chemiluminescent immunometric method LIAISON (DiaSorin, Saluggia, Italy) applied to a fully automated analyzer was used for measuring direct renin concentration (DRC) and aldosterone. Intra-assay variation was less than 7.2% over the range 25–107 mIU/L, and inter-assay variation was less than 10.4% over the range 4.9–110 mIU/L for DRC (the functional sensitivity was below 2.0 mIU/L, and the limit of detection was 0.33 mIU/L). Regarding aldosterone, pool samples and controls over the range 68–749 ng/L yielded intra- and inter-assay coefficient of variations of 1.8–4.2% and 5.6–10.5%, respectively. This assay had a wide measurement range varying from 9.7 ng/L up to 1000 ng/L. The functional sensitivity was 19.7 ng/L.

Serum creatinine, glucose, total cholesterol, HDL-cholesterol, and triglycerides levels (mg/dL) were measured by enzymatic colorimetric method (AU5800, Beckman Coulter Inc, USA). HbA1c (mmol/mol) were measured by method D 100 HbA HPLC (BioRad Lab, USA). Serum Na (mmol/L) and K (mmol/L) were evaluated with impedance measured method (AU5800, Beckman Coulter Inc, USA). P-Osm (mOsm/kg) and u-Osm (mOsm/kg) were measured by automatic osmometer (Osmo Station OM 6050, ARKRAY Global, Kyoto, Japan) using freezing point depression method.

### Statistical analysis

Categorical data were expressed as absolute or relative frequencies (n, %). Continuous variables were reported as median [25th -75th percentile] or mean ± standard deviation (SD), according to their distribution, evaluated with Kolmogorov-Smirnov test. Comparison of continuous variables between groups was performed by T Student test or ANOVA for independent samples in case of normal distribution, or by Mann-Whitney U test or Kruskal-Wallis test if not normally distributed. Chi-square test or Fisher’s exact test were used for categorical variables, as appropriate. Wilcoxon signed-rank test was used for the analysis of paired samples. The correlation between continuous variables was evaluated with Pearson test. Group classification based on AUC_E0→E5 percentiles and between-group comparisons were defined a priori. Correlation analyses were exploratory and interpreted accordingly. Effect sizes were considered to aid interpretation of key findings. Since this study could be considered as proof-of-concept, we didn’t perform a quantification of the statistical power and we didn’t define a minimum sample size. Two-sided *p* < 0.05 was considered significant. Data analysis was performed by GraphPad Prism (version 10.5.0; GraphPad Software Inc., CA, USA).

## Results

### General characteristics of PAI patients

Nineteen adults with primary adrenal insufficiency (PAI) were enrolled, with a balanced sex distribution (47.4% males, 52.6% females) and a mean age of 47.9 ± 12.7 years (range 21–70). Six patients had isolated autoimmune Addison’s disease, twelve had autoimmune polyendocrine syndromes (APS) of various subtypes (APS-2 = 10, APS-1 = 1, APS-4 = 1), and one had idiopathic PAI. Within the APS cohort, eight patients had autoimmune primary hypothyroidism (adequately treated with levothyroxine) and one had primary hypoparathyroidism (treated with calcium carbonate and calcitriol with good control). Additional endocrine and non-endocrine autoimmune disorders are detailed in Table 1. Five patients had diabetes mellitus (three type 1 and two type 2). All were treated with fludrocortisone (median dose 0.075 [0.05–0.10] mg/day) and HC: eight on conventional immediate-release (IR-HC; median 20 [20-20] mg/day), eleven on dual-release (DR-HC; median 20 [20-20] mg/day), and one on DR-HC 20 mg plus IR-HC 10 mg/day. No patients received cortisone acetate. IR-HC was administered two or three times daily.

**Table 1:** General characteristics of PAI patients

Case	Sex	Age (years)	Aetiology	Associated diseases	Disease duration (years)	GC therapy (mg/daily)	MC therapy (mg/daily)
1	M	54	Idiopathic	HYPOTH	14	DR-HC 25	0.05
2	F	42	APS2	HYPOTH, CD	14	DR-HC 15	0.1
3	F	34	APS1	HYPOPTH, POI, Al	28	DR-HC 20	0.05
4	M	46	APS2	HT	36	DR-HC 20	0.15
5	F	21	AI	/	4	DR-HC 20	0.125
6	M	64	APS4	VIT, CAG	25	DR-HC 20	0.1
7	M	33	AI	HYPOTH	15	DR-HC 20	0.05
8	F	57	APS2	HT	18	DR-HC 20	0.05
9	F	52	APS2	HYPOTH, DMT1, POI	13	HC 20	0.15
10	F	54	APS2	HYPOTH, POI	6	DR-HC 20 + HC 10	0.05
11	M	62	AI	DMT2	23	DR-HC 25	0.1
12	F	57	APS2	HYPOTH, DM1	15	DR-HC 20	0.05
13	M	47	AI	/	11	HC 20	0.1
14	M	36	AI	/	18	DR-HC 20	0.075
15	M	61	APS2	HYPOTH	11	HC 20	0.075
16	F	45	AI	/	3	HC 20	0.1
17	M	38	APS2	HT, AT	7	HC 20	0.1
18	F	70	APS2	HYPOTH, CAG, DMT2	17	HC 20	0.05
19	F	38	APS2	DMT1, CAG	4	HC 17.5	0.05

Abbreviations: M, male; F, female; APS, autoimmune polyendocrine syndrome; AI, autoimmune isolated; HYPOTH, hypothyroidism; CD, coeliac disease; HYPOPTH, hypoparathyroidism; POI, primary ovary insufficiency; Al, alopecia; HT, Hashimoto’s thyroiditis; VIT, vitiligo; CAG, chronic atrophic gastritis; DMT1, type 1 diabetes mellitus; DMT2, type 2 diabetes mellitus; AT, autoimmune thrombocytopenia; GC, glucocorticoid; MC, mineralocorticoid

Anthropometric and haemodynamic parameters were within normal ranges, including weight, BMI, waist circumference, blood pressure, and heart rate. Among biochemical parameters, electrolytes were normal both in serum (s-Na 139.1 ± 4.50 mmol/L; s-K 4.13 ± 0.39 mmol/L) and spot urine (u-Na 115.7 ± 44.8 mmol/L; u-K 29.9 ± 13.5 mmol/L). Plasma and urinary osmolality were 283.6 ± 8.0 mOsm/kg and 613.9 ± 226.7 mOsm/kg, respectively. Renal function was preserved and mean LDL-cholesterol was 95.3 ± 29.1 mg/dL.

Health-related quality of life (HRQoL), assessed using the AddiQoL-30 questionnaire, showed a mean total score of 91.9 ± 8.9/120, with a mean of 4.6 ± 3.0 pathological items per subject.

Baseline hormonal assessment before morning replacement therapy demonstrated median renin and copeptin values within the reference range, whereas ACTH concentrations exceeded the upper normal limit.

**Fig. 1:**
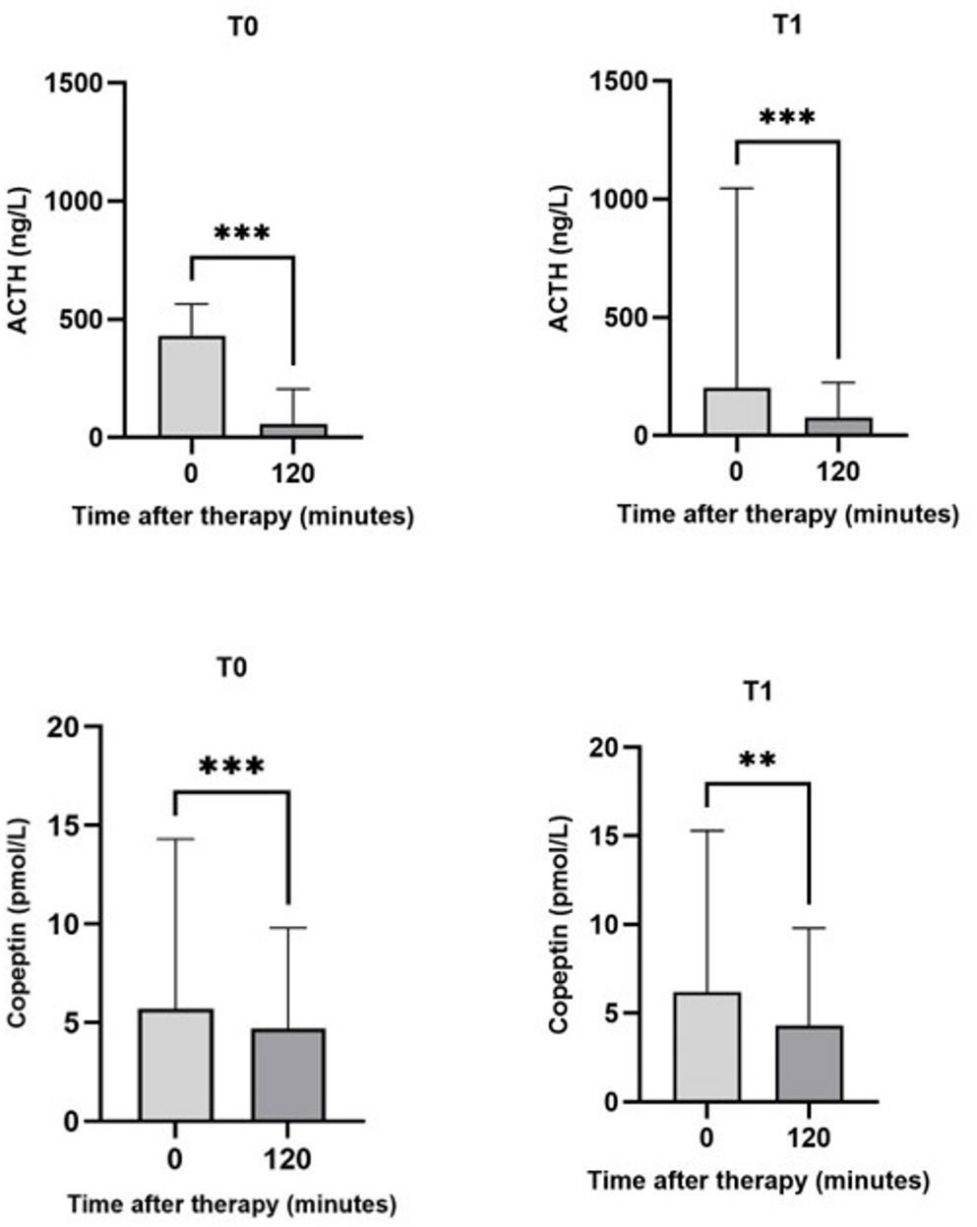
ACTH and copeptin levels before and 120 min after replacement therapy at T0 and T1. Data are expressed as median and interquartile range. ** *p* < 0.01, *** *p* < 0.001

After HC intake, both at baseline (T0) and follow-up (T1), ACTH and copeptin decreased significantly (Fig. 1). At T0, ACTH fell from 439.4 [154.6–562.3] ng/L to 56.2 [45.4–196.0] ng/L (*p* < 0.001) and copeptin from 6.10 [4.73–13.18] pmol/L to 4.70 [3.90–9.80] pmol/L (*p* < 0.001), with no significant change in renin. The same pattern was confirmed at T1: ACTH decreased from 200.1 [122.2–1046] ng/L to 75.4 [35.9–224.9] ng/L (*p* < 0.001) and copeptin from 8.30 [5.00–15.30] pmol/L to 5.50 [3.80–9.80] pmol/L (*p* = 0.001), while renin remained stable. These data are summarized in Table 2.

**Table 2:** ACTH, renin and copeptin levels before and 120 min after replacement therapy at T0 and T1

	T0			T1		
	0’	120’	*p*-value	0’	120’	*p*-value
ACTH (ng/L)	439.4 [154.6-562.3]	56.20 [45.40–196.0]	*< 0.001*	200.1 [122.2–1046]	75.40 [35.90-224.90]	*< 0.001*
Renin (mUI/L)	33.37 [12.67–13.18]	24.93 [13.12–81.59]	0.229	58.30 [23.30–99.90]	52.10 [4.30–88.90]	0.347
Copeptin (pmol/L)	6.10 [4.73–13.18]	4.70 [3.90–9.80]	*< 0.001*	8.30 [5.00-15.30]	5.50 [3.80–9.80]	*0.001*

Data are expressed as median [25th -75th percentile]

## Assessment of GC replacement therapy

Both salivary cortisol and cortisone AUCs were characterized by high variability; in particular, AUC_F0→F5 showed a coefficient of variation (CV) of 97%, while the CV of AUC_E0→E5 was lower (36%).

Moreover, salivary cortisol concentrations displayed a steeper decrease approximately 8 h and 30 min after therapy, whereas salivary cortisone levels remained more stable throughout the observation period. Both salivary cortisol and cortisone values showed a marked peak about 1 h and 30 min after hydrocortisone (HC) intake.

To assess the adequacy of GC replacement therapy in patients with PAI, we compared their cortisone AUC values with those of healthy controls. Based on this comparison, PAI patients were classified into three groups:

- Group A (likely overtreated): AUC_E0→E5 values above the 90th percentile of the control group;
- Group B (adequate therapy): AUC_E0→E5 values between the 10th and 90th percentiles of the control group;
- Group C (likely undertreated): AUC_E0→E5 values below the 10th percentile of the control group.

In our cohort, the 90th and 10th percentile values of the control group’s AUC_E0→E5 were 21.521 and 8.768, respectively. At baseline (T0), six PAI patients (31.6%) had AUC_E0→E5 values above the 90th percentile, indicating probable GC overtreatment (Group A). At follow-up (T1), this proportion increased to seven patients (36.8%). The remaining patients—13 at T0 (68.4%) and 12 at T1 (63.2%)—fell within the 10th − 90th percentile range, indicating adequate GC replacement (Group B). No patients had AUC_E0→E5 values below the 10th percentile, and thus none were classified as undertreated (Group C).

Subsequently, we examined the distribution of anthropometric, biometric, biochemical, and hormonal parameters across these two groups of patients. As shown in Table 3, no substantial differences were observed in anthropometric or biometric characteristics, except for blood pressure profile at T0. Specifically, diastolic blood pressure (DBP) values were significantly higher (*p* = 0.042) in Group A (82.00 ± 7.58 mmHg) compared with Group B (73.85 ± 6.82 mmHg). Conversely, differential blood pressure values were significantly lower (*p* = 0.018) in Group A (31.00 ± 7.42 mmHg) than in Group B (41.92 ± 8.05 mmHg). Moreover, Group A and Group B did not differ concerning AddiQol.

**Table 3:** Anthropometric and biometric parameters in all PAI patients, group A (GC excess) and group B (non-GC excess) at T0 and T1

	T0				T1			
	All patients (*n* = 19)	Group A (*n* = 6)	Group B (*n* = 13)	*p*-value A vs. B	All patients (*n* = 19)	Group A (*n* = 7)	Group B (*n* = 12)	*p*-value A vs. B
**Age** **(years)**	47.95 ± 12.69	50.83 ± 13.33	46.62 ± 12.71	0.516	48.95 ± 12.69	52.14 ± 10.99	47.08 ± 13.69	0.418
**Male sex** **(n**,** %)**	9, 47.4	1, 16.7	8, 61.5	0.141	9, 47.3	2, 28.6	7, 58.3	0.350
**Weight** **(kg)**	64.26 ± 11.17	62.17 ± 9.30	65.23 ± 12.17	0.593	64.00 ± 10.48	63.71 ± 11.60	64.17 ± 10.31	0.931
**Height** **(m)**	1.65 ± 0.07	1.60 ± 0.04	1.67 ± 0.07	0.063	1.65 ± 0.07	1.63 ± 0.04	1.66 ± 0.08	0.557
**BMI** **(kg/m2)**	23.46 ± 3.45	24.17 ± 3.49	23.14 ± 3.53	0.561	23.53 ± 3.30	23.73 ± 3.47	23.41 ± 3.34	0.842
**Waist** **(cm)**	81.50 ± 9.15	75.96 ± 7.16	83.81 ± 9.14	0.109	82.78 ± 11.18	87.25 ± 11.13	79.93 ± 10.73	0.183
**HR** **(bpm)**	68.21 ± 8.29	63.33 ± 4.50	70.46 ± 8.78	0.081	68.37 ± 6.40	67.86 ± 5.52	68.67 ± 7.08	0.799
**SBP** **(mmHg)**	115.0 ± 10.57	113.0 ± 6.71	115.8 ± 11.88	0.633	114.0 ± 13.08	117.7 ± 10.80	111.8 ± 14.15	0.357
**DBP** **(mmHg)**	76.11 ± 7.78	82.00 ± 7.58	73.85 ± 6.82	*0.042*	75.68 ± 9.30	80.86 ± 6.59	75.76 ± 9.53	0.062
**Diff. BP** **(mmHg)**	38.89 ± 9.16	31.00 ± 7.42	41.92 ± 8.05	*0.018*	38.32 ± 9.13	36.86 ± 6.26	39.17 ± 10.62	0.609
**HC** **(mg/day)**	20.00 [20.00–20.00]	20.00 [18.75–21.25]	20.00 [20.00–20.00]	0.732	20.00 [20.00–20.00]	20.00 [20.00–25.00]	20.00 [20.00–20.00]	0.243
**HC/BSA** **(mg/m2)**	12.00 [10.63–13.23]	11.85 [10.74–13.51]	12.00 [10.45–13.34]	0.964	12.14 [10.75–13.89]	12.60 [10.63–16.55]	12.09 [10.83–13.20]	0.596
**FC** **(mg/day)**	0.10 [0.05–0.10]	0.10 [0.07–0.11]	0.08 [0.05–0.10]	0.333	0.10 [0.05–0.10]	0.08 [0.05–0.10]	0.10 [0.06–0.10]	0.767

Data are reported as median [25th-75th percentile] or mean ±standard deviation, and as frequency count, as appropriate

Abbreviations: BMI, body mass index; HR, heart rate; SBP, systolic blood pressure; DBP, diastolic blood pressure; Diff. BP, differential blood pressure; HC, hydrocortisone; BSA, body surface area. FC, fludrocortisone

The analysis of biochemical parameters is summarized in Table 4. The two groups differed significantly with regard to glucose metabolism. Median HbA1c levels were higher in Group A than in Group B at T0 (46.50 [36.00–53.50] mmol/mol vs. 34.00 [30.50–36.50] mmol/mol, respectively; *p* = 0.026). This difference persisted at T1 (51.00 [40.00–62.00] mmol/mol vs. 33.50 [31.25–36.25] mmol/mol; *p* = 0.002) and remained significant after excluding diabetic patients from the analysis. Electrolyte levels, renal function, and both plasma and urine osmolality were comparable between the two groups.

**Table 4:** Biochemical parameters in all PAI patients, group A (GC excess) and group B (non-GC excess) at T0 and T1

	T0				T1			
	All patients (*n* = 19)	Group A (*n* = 6)	Group B (*n* = 13)	*p*-value A vs. B	All patients (*n* = 19)	Group A (*n* = 7)	Group B (*n* = 12)	*p*-value A vs. B
**HbA1c (mmol/mol)**	36.00 [33.00–48.00]	46.50 [36.00-53.50]	34.00 [30.50–36.50]	*0.026*	34.00 [32.00–51.00]	51.00 [40.00–62.00]	33.50 [31.25–36.25]	*0.002*
**Glucose** **(mg/dl)**	76.00 [67.00–88.00]	84.50 [69.75–94.75]	76.00 [66.00-82.50]	0.480	79.00 [70.00-100.0]	100.0 [80.00-195.0]	75.00 [68.00-79.75]	*0.003*
**Total cholesterol (mg/dl)**	188.4 ± 24.20	181.6 ± 20.06	191.5 ± 26.02	0.425	215.3 ± 26.02	211.0 ± 14.59	217.8 ± 31.20	0.600
**HDL** **(mg/dl)**	72.00 ± 21.48	88.00 ± 15.13	64.62 ± 20.24	*0.023*	67.26 ± 16.18	77.71 ± 16.42	61.17 ± 13.08	*0.027*
**TG** **(mg/dl)**	105.3 ± 54.97	90.17 ± 35.99	112.3 ± 61.85	0.430	100.3 ± 50.47	89.71 ± 45.13	106.5 ± 13.08	0.500
**cLDL** **(mg/dl)**	95.29 ± 29.11	83.40 ± 7.83	100.8 ± 29.07	0.237	132.8 ± 30.51	129.3 ± 29.32	135.0 ± 32.45	0.713
**s-Na** **(mmol/L)**	139.1 ± 4.50	138.7 ± 4.08	139.2 ± 4.83	0.808	139.1 ± 2.62	139.3 ± 3.09	139.0 ± 2.45	0.826
**s-K** **(mmol/L)**	4.13 ± 0.39	3.98 ± 0.47	4.20 ± 0.34	0.267	4.04 ± 0.27	4.09 ± 0.13	4.02 ± 0.33	0.610
**Creatinine (mg/dl)**	0.81 [0.71–0.95]	0.85 [0.78–1.09]	0.76 [0.69–0.92]	0.146	0.87 [0.70–0.97]	0.87 [0.73–1.12]	0.83 [0.69–0.97]	0.576
**p-Osm (mOsm/kg)**	283.6 ± 8.03	285.8 ± 6.91	282.7 ± 8.57	0.482	286.7 ± 6.26	287.7 ± 4.79	286.0 ± 7.18	0.236
**u-Osm (mOsm/kg)**	613.9 ± 226.7	658.6 ± 92.99	595.3 ± 265.2	0.616	598.9 ± 215.1	505.1 ± 202.2	658.6 ± 209.3	0.145

Data are reported as median [25th-75th percentile] or mean ± standard deviation, and as frequency count, as appropriate

Abbreviations: Na, sodium; K, potassium, p-Osm, plasmatic osmolality; u-Osm, urine osmolality

A difference was also observed in lipid profile. HDL-cholesterol concentrations were higher in Group A both at T0 (88.00 ± 15.13 mg/dL vs. 64.62 ± 20.24 mg/dL; *p* = 0.023) and at T1 (77.71 ± 16.42 mg/dL vs. 61.17 ± 13.08 mg/dL; *p* = 0.027).

Table 5 shows the hormonal parameters measured both at baseline and 120 min after replacement therapy in relation to therapeutic adequacy. Group A and Group B differed significantly in several hormonal values. At T0, median ACTH levels were lower in Group A compared with Group B, both at baseline (146.7 [13.53–155.5] ng/L vs. 552.7 [383.6–942.7] ng/L; *p* = 0.001) and 120 min after therapy (35.80 [5.33–52.43] ng/L vs. 157.8 [55.10–324.2] ng/L; *p* = 0.001). This difference persisted at T1, with lower median ACTH values in Group A both before (122.6 [51.40–182.6] vs. 528.6 [185.3–1131] ng/L; *p* = 0.045) and after therapy (35.90 [14.80–51.00] vs. 154.8 [56.20–367.9] ng/L; *p* = 0.028).

**Table 5:** Hormonal parameters in all PAI patients, group A (GC excess) and group B (non-GC excess) at T0 and T1

	T0				T1			
	All patients (*n* = 19)	Group A (*n* = 6)	Group B (*n* = 13)	*p*-value A vs. B	All patients (*n* = 19)	Group A (*n* = 7)	Group B (*n* = 12)	*p*-value A vs. B
**ACTH** **(ng/L)**	431.4 [154.3-564.3]	146.7 [13.53–155.5]	552.7 [383.6-942.7]	*0.001*	200.0 [122.2–1046]	122.6 [51.40-182.6]	528.6 [185.3–1131]	*0.045*
**Renin (mUI/L)**	25.40 [12.55–41.17]	31.04 [9.89–47.17]	25.40 [12.75–119.7]	0.765	58.50 [23.30–99.90]	30.50 [6.00-61.30]	67.40 [47.83–111.4]	0.227
**Copeptin** **(pmol/L)**	5.70 [4.70–14.30]	16.60 [4.30-24.48]	5.40 [4.25–9.30]	0.282	6.20 [5.00-15.30]	7.25 [3.33–15.75]	6.10 [5.40–12.10]	0.837
**Cop/u-Na**	7.04 [3.79–12.95]	12.75 [3.49–22.80]	5.94 [3.64–11.12]	0.368	8.24 [4.18–12.25]	10.43 [8.47–14.59]	4.46 [2.99–8.24]	*0.044*
**ACTH/Cop**	58.89 [22.47–170.5]	6.41 [1.30-44.46]	109.1 [40.73–198.2]	*0.013*	54.61 [16.71–165.5]	22.63 [3.14–29.93]	82.00 [36.90-169.6]	*0.044*
**Renin/Cop**	4.49 [0.86–11.21]	2.68 [0.41–15.20]	4.70 [2.05–12.66]	0.521	6.28 [1.11–21.23]	2.56 [0.60-12.26]	7.11 [4.12–21.88]	0.261
**ACTH 120’** **(ng/L)**	56.20 [45.40–196.0]	35.80 [5.33–52.43]	157.8 [55.10-324.2]	*0.001*	75.40 [35.90-224.9]	35.90 [14.80–51.00]	154.8 [56.20-367.9]	*0.028*
**Renin 120’** **(mUI/L)**	24.93 [13.12–81.59]	25.05 [9.40-82.66]	24.93 [13.41–98.65]	0.579	52.10 [4.30–88.90]	35.80 [3.50–69.10]	52.35 [15.00-91.15]	0.592
**Copeptin 120’** **(pmol/L)**	4.70 [3.90–9.80]	11.40 [3.38–20.23]	4.70 [3.75–7.25]	0.535	7.00 [3.80–9.80]	8.50 [4.20–12.50]	4.30 [3.15–9.63]	0.290
**ACTH/Cop 120’**	13.70 [8.65–50.36]	2.13 [1.08–20.74]	33.57 [10.94–65.75]	*0.035*	7.08 [1.06–21.23]	2.86 [0.90-16.45]	9.77 [3.78–23.05]	0.104
**Renin/Cop 120’**	7.88 [1.78–15.88]	9.36 [0.59–44.37]	7.41 [2.71–14.34]	0.849	18.55 [6.33–37.69]	5.41 [1.17–12.14]	34.43 [16.60-59.18]	*0.003*

Data are reported as median [25th-75th percentile] or mean ± standard deviation, as appropriate

Abbreviations: Cop, copeptin; Na, sodium

**Fig. 2:**
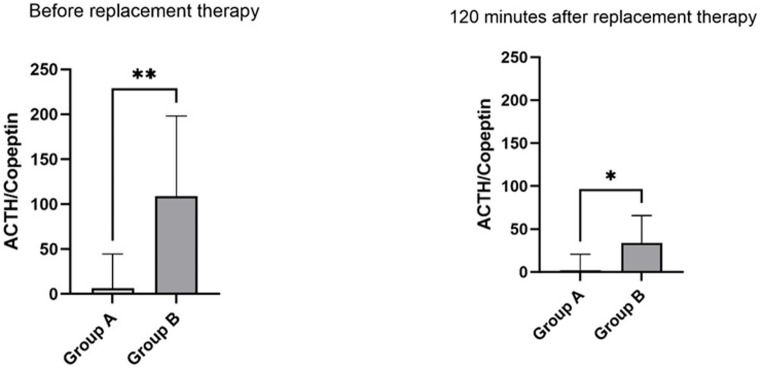
ACTH/Copeptin ratio evaluated in Group A (GC excess) and Group B (non-GC excess) before and 120 min after replacement therapy at T0. Data are expressed as median and interquartile range. *p < 0.05, ** *p* < 0.01

As shown in Fig. 2, Group A and Group B also differed significantly in the ACTH/copeptin ratio, which was lower in patients with GC excess both before therapy (6.41 [1.30–44.46] vs. 109.1 [40.73–198.2]; *p* = 0.013) and 120 min after replacement therapy (2.13 [1.08–20.74] vs. 33.57 [10.94–65.75]; *p* = 0.035). This difference was confirmed at T1 for baseline ACTH/copeptin values.

Indeed ACTH/copeptin ratio showed an inverse correlation with cortisone AUC (*r* = − 0.59, *p* < 0.01).

**Fig. 3:**
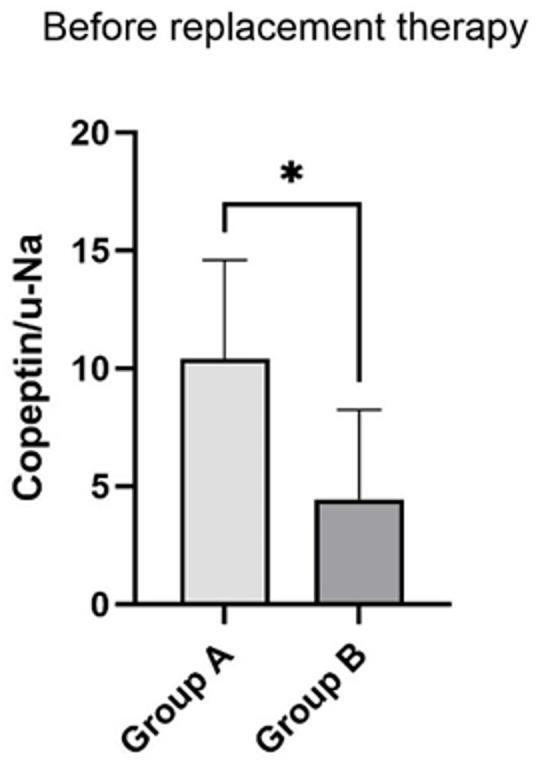
Copeptin/u-Na ratio evaluated in Group A (GC excess) and Group B (non-GC excess) before replacement therapy at T1. Data are expressed as median and interquartile range. *p < 0.05

**Fig. 4:**
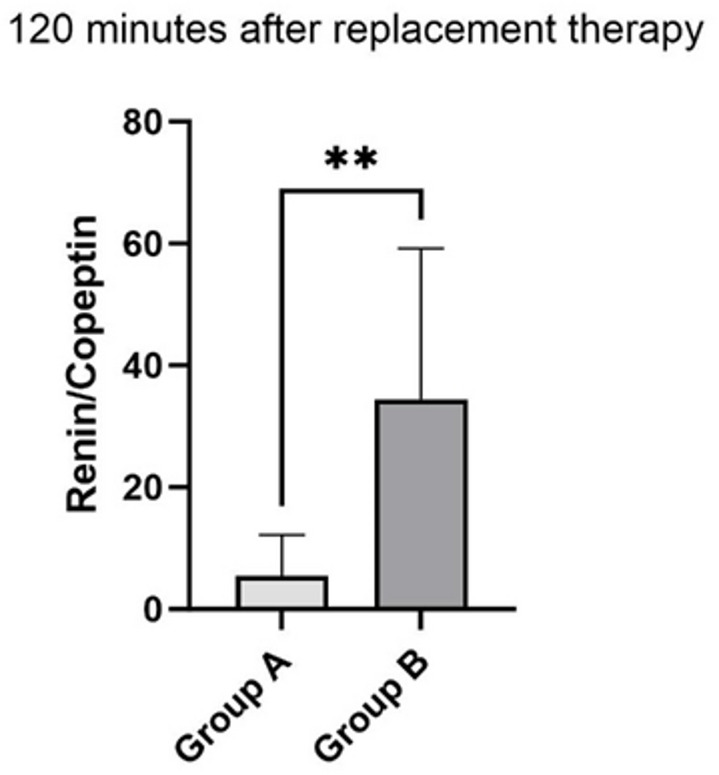
Renin/copeptin ratio evaluated in Group A (GC excess) and Group B (non-GC excess) 120 min after replacement therapy at T1. Data are expressed as median and interquartile range. ** *p* < 0.01

We also found a significant difference between the two groups regarding the copeptin/u-Na ratio at T1, with higher median values in Group A than in Group B (10.43 [8.47–14.59] vs. 4.46 [2.99–8.24]; *p* = 0.044), as shown in Fig. 3. Furthermore, at T1, the renin/copeptin ratio measured 120 min after replacement therapy showed a different distribution between the groups, being lower in patients with GC excess (5.41 [1.17–12.14] vs. 34.43 [16.60–59.18]; *p* = 0.003), as reported in Fig. 4.

## Discussion

In the present study we aimed to explore the potential role of novel biochemical markers in the therapeutic monitoring of patients affected by PAI. We quantified the extent of GC exposure through the comparison of salivary cortisone AUC values detected in PAI patients with those obtained from healthy controls, identifying a subgroup of patients in likely GC excess and another in adequate replacement. The distribution of anthropometric, biochemical, metabolic, and hormonal variables was then analyzed across these groups.

The main findings demonstrated that patients classified as being in GC excess were characterized by (a) poorer glucose metabolism, independent of diabetes status; (b) altered blood pressure profile; (c) reduced ACTH levels and lower ACTH/copeptin ratios both before and 120 min after therapy intake; (d) higher baseline copeptin/u-Na ratios; and (e) lower renin/copeptin ratios measured post-dose.

We observed that the association regarding glycemic status defined by HbA1c, ACTH levels and ACTH/copeptin ratio maintained their statistical significance also during follow-up, so we can postulate that these parameters may represent more robust markers of chronic GC overexposure.

As reported in the literature, the major limitations of GC replacement therapy are the inability to reproduce the physiological circadian rhythm of cortisol secretion and the lack of reliable biochemical tools for therapy monitoring [4, 5, 20, 44]. Mah et al. [45] proposed a nomogram based on serum cortisol concentrations 2.5–5 h after GC administration compared with reference percentiles, as a means for dose adjustment. However, this approach is cumbersome, costly, and unsuitable for outpatients, as it requires multiple venipunctures. Consequently, salivary cortisol day-curves have been proposed as an alternative monitoring strategy, given that saliva sampling is non-invasive, stress-free, and easily repeatable [12, 22]. Although salivary and serum cortisol concentrations generally correlate well, the high variability of salivary cortisol limits its clinical utility for dose titration [46].

Ross et al. [47] observed significantly higher salivary cortisol AUCs in 31 patients with Addison’s disease receiving hydrocortisone (HC) replacement compared to healthy controls. Similarly, Ceccato et al. [13], studying 18 patients with Addison’s disease and 43 controls, found that salivary cortisol not only reflected the cortisol profile but also captured pharmacokinetic differences between formulations, showing lower afternoon–evening exposure in dual-release HC (DR-HC) compared with immediate-release (IR-HC). Increasing evidence suggests, however, that salivary cortisone may provide a superior reflection of serum cortisol levels [24]. In serum, cortisol concentrations exceed cortisone at an approximate 4:1 ratio, whereas in saliva this ratio is reversed (> 4:1 cortisone: cortisol) due to abundant 11β-hydroxysteroid dehydrogenase type 2 activity in salivary glands. Consequently, salivary cortisone closely mirrors serum cortisol and can serve as its surrogate marker.

In the prospective cross-over study by Debono et al. [25], conducted in 14 volunteers, salivary cortisone more accurately tracked cortisol circadian rhythm under both physiological conditions and oral HC replacement, without the contamination issues seen with salivary cortisol.

On these grounds, we selected salivary cortisone AUCs for comparison with healthy subjects to evaluate GC exposure in PAI. In our cohort, cortisol AUCs displayed greater dispersion and fell below detection at later time points, while cortisone values remained measurable throughout the day, supporting their use as a robust biomarker of systemic exposure. Importantly, all enrolled patients were on hydrocortisone therapy—either IR or DR—making them suitable for evaluation via salivary cortisone profiling [24, 25]. Replacement regimen heterogeneity (IR-HC vs. DR-HC) may influence early post-dose cortisol exposure and subsequent hormonal feedback; however, given limited subgroup sizes, formulation-stratified or adjusted analyses would yield unstable estimates and were not pursued. Larger prospective cohorts should stratify by hydrocortisone formulation and incorporate regimen type in adjusted models.

No patients exhibited cortisone AUC below the 10th percentile of controls, indicating an absence of undertreatment. This may reflect the clinical imperative to avoid under-replacement in PAI, which could precipitate adrenal crisis and worsen quality of life. As a result, mild over-replacement may occur in some patients, often going unrecognised and traditionally considered clinically insignificant [8]. However, accumulating evidence now links chronic GC overexposure to increased cardiovascular and skeletal risk, impaired quality of life, and higher mortality [9].

We highlight that the percentile-based definition of GC excess is exploratory; prospective validation using pre-specified, outcome-driven thresholds (e.g., ROC analyses against clinical endpoints) is warranted.

Our findings reinforce the well-established effects of GC excess on glucose metabolism: higher HbA1c and fasting glucose values were observed in overtreated patients, irrespective of diabetes mellitus type 1 (DMT1) status. In this setting, excessive GC replacement may further impact insulin requirements and glycaemic control. We also found an association between GC excess and elevated diastolic blood pressure with reduced pulse pressure. However, we could hypothesize that HbA1c reflects longer-term exposure and may therefore be a more stable marker of chronic overexposure, whereas single-visit blood pressure measures may be more sensitive to day-to-day variability (stress, activity, salt/fluid intake, measurement conditions), particularly in a small cohort.

Moreover, the impact of GC therapy on blood pressure in PAI remains controversial, as recent data suggest that genetic background may modulate hypertension risk more than HC dose [48]. Conversely, other studies have shown that higher HC doses increase both systolic and diastolic pressure, whereas switching from IR-HC to DR-HC may improve haemodynamic parameters [11, 15].

Regarding lipid metabolism, patients with GC excess showed higher HDL-cholesterol levels, consistent with previous reports that GCs exert complex and context-dependent effects on lipolysis, free fatty acid turnover, and lipid trafficking. A subtle difference in sex distribution—slightly more females in the GC-excess group—may partially account for this finding, as HDL-cholesterol is typically higher in women.

As expected, GC excess correlated with lower ACTH concentrations both before and after replacement therapy. Literature consistently shows that suppressed or normal ACTH values indicate potential over-replacement, whereas elevated ACTH should not automatically prompt dose escalation [4]. The limited accuracy of ACTH as a monitoring biomarker may stem from reduced pituitary sensitivity to cortisol inhibition or, more plausibly, from the inability of conventional HC regimens to sustain physiological cortisol levels over 24 h.

Copeptin, a stable surrogate of arginine vasopressin (AVP), has emerged as a reliable biomarker of antidiuretic system activity [30, 49]. It has diagnostic and prognostic utility in cardiovascular disease [31, 32, 50] and electrolyte disorders [35, 51]. However, to our knowledge, its role in the management of PAI—a paradigmatic condition of combined GC and mineralocorticoid deficiency—has not been previously explored.

From a physiological standpoint, AVP secretion is mainly regulated by the effective osmotic pressure of the extracellular body fluid, due to the activity of osmoregulatory hypothalamic neurons, and by more pronounced decreases in extracellular volume. A second neurosecretory pathway transports AVP from the hypothalamic parvocellular neurons to the pituitary portal system where AVP acts synergistically with CRH to stimulate ACTH release in response to stress. Additionally, there is evidence that GCs could suppress AVP secretion via negative feedback.

Therefore, copeptin measurement may provide additional insight into hypothalamic–pituitary–adrenal (HPA) axis activity during replacement therapy. Since AVP/copeptin secretion responds also to osmotic and haemodynamic stimuli, mineralocorticoid treatment may further influence this relationship.

In our study, copeptin tended to be higher in patients with GC excess, though not significantly so. When considered relative to urinary sodium, the copeptin/u-Na ratio was significantly increased in overexposed patients, in agreement with evidence that this index can distinguish volume-depleted from normovolaemic hyponatraemic states [52]. More strikingly, ACTH/copeptin ratios were consistently lower in GC-excess patients at baseline and post-dose at enrollment, and at baseline after 12 months, suggesting enhanced feedback inhibition of both ACTH and AVP pathways under GC overexposure. The between-group separation in ACTH/copeptin appears largely attributable to ACTH suppression. Accordingly, ACTH/copeptin is presented as a contextual, hypothesis-generating composite index rather than a surrogate endpoint. In contrast, the renin/copeptin ratio—measured 120 min after therapy—was reduced in the GC-excess group at follow-up, possibly reflecting the complex and multifactorial regulation of renin, influenced by drug therapy, sodium intake, and volume status.

The higher copeptin levels observed in GC-excess patients may relate to the slightly higher plasma sodium and osmolarity observed in this group, although these parameters did not reach statistical significance. The marked post-dose reduction in copeptin further supports its sensitivity to acute glucocorticoid feedback and compensatory fluid balance mechanisms. Collectively, these results reinforce the concept that current GC regimens fail to fully mimic the physiological circadian rhythm, leading to exaggerated morning peaks and absent nocturnal nadirs, which may result in lower copeptin and higher ACTH relative to healthy individuals.

Nevertheless, potential confounding factors affecting copeptin, such as hydration status, sodium intake, stress, and haemodynamics, should be considered in the interpretation of these results. For these reasons, in our framework, copeptin is not proposed here as a standalone biomarker of GC adequacy; rather, we explored copeptin primarily through ratio-based indices (ACTH/copeptin; renin/copeptin; copeptin/u-Na) to contextualise ACTH feedback within AVP-related osmotic and haemodynamic regulation.

Concerning blood pressure profile and the related hormonal parameters, we underlined an apparent dissociation in the results. As we found higher diastolic blood pressure in the overtreated patients, we also expected a significant variation in renin and copeptin levels, because, for a physiological standpoint, changes in blood pressure and effective circulating volume are typically integrated through coordinated modulation of the renin-angiotensis-aldosterone system (RAAS) and AVP system. As a possible explanation, we can postulate that in PAI, integrated RAAS/AVP readouts may be blunted or masked by (i) GC-mediated vascular effects and sympathetic tone, (ii) MC replacement and salt intake variability, (iii) copeptin’s strong osmotic and stress responsiveness, and (iv) timing of sampling relative to dose and daily activities.

Furthermore, for the above-mentioned significant variability of single-visit blood pressure measures, we aimed to validate in future studies haemodynamic findings using evaluation of ambulatory BP profile.

In our study we did not observe a difference in self-perceived quality of life based on GC replacement status, as a possible consequence of limited power of the study and short timeframe relative to chronic complications.

The main strengths of this study include its cross-sectional case–control design, the analytical robustness of LC–MS/MS steroid quantification, and the replication of findings after 12 months of stable treatment.

However, our study presents some limitations. First, the small sample size, intrinsic to the rarity of PAI and to the strict inclusion criteria (only HC-treated patients) and the absence of individuals below the 10th percentile of control AUC_E0→E5 (i.e., no undertreated group) limits generalizability, underscoring the need for larger prospective studies including patients across the full spectrum of replacement adequacy. Secondly, as a proof-of-concept study, we did not perform a formal power calculation; therefore, negative findings should be interpreted cautiously. In addition, both GC regimen (IR-HC vs. DR-HC) and variability in fludrocortisone dosing may influence ACTH suppression, haemodynamic parameters, renin, and copeptin. Given limited sample size, stratified or adjusted sensitivity analyses would be statistically unstable; larger prospective studies should address these confounders by design. Moreover, adherence to GC and MC therapy was not objectively assessed; although patients were on stable regimens and followed a structured sampling protocol, suboptimal adherence may have influenced ACTH, renin and copeptin-derived indices. Furthermore, the study did not evaluate some hard clinical outcomes of GC adequacy, such as cardiovascular events, fragility fractures or adrenal crisis. Finally, copeptin measurement is not universally available, and renin evaluation is affected by many pre-analytical (posture, salt intake, volume status and interfering drugs) and analytical (assay-related) critical issues. These criticisms could limit the immediate translational applicability of our findings.

Until now, the assessment of treatment adequacy in PAI has largely relied on clinical judgement, symptom evaluation, and patient-reported well-being [4, 5, 20, 42]. The identification of objective biochemical parameters is therefore a key unmet need. Our results indicate that salivary cortisone profiling—particularly when benchmarked to control percentiles—could capture glucocorticoid overexposure, which correlates with recognised GC-related metabolic and cardiovascular complications. The observed associations with HbA1c and blood pressure strengthen its clinical relevance. Moreover, copeptin and the derived ACTH/copeptin and renin/copeptin ratios could be candidate adjunct biomarkers for refining therapy evaluation, while other studies are required to validate these preliminary observations. From the clinical standpoint, this study might provide new insights in the management of GC replacement therapy in PAI, despite the previously mentioned limitations concerning their application in routine clinical practice. On the other hand, for research purposes, it could be interesting to investigate the response of copeptin to HC administration independently of fludrocortisone intake.

This proof-of-concept study suggests salivary cortisone AUC, together with ACTH/copeptin and renin/copeptin ratios, as pragmatic, physiology-informed candidates for monitoring GC replacement in PAI, with particular emphasis on ratio-based indices integrating GC exposure and neuroendocrine feedback. Validation in larger, prospective cohorts with pre-specified thresholds and adjustment for hydrocortisone formulation is warranted.

## Supplementary Information

Below is the link to the electronic supplementary material.

Supplementary Material 1
