# Supplementary material for: Novel biomarkers to guide glucocorticoid replacement in primary adrenal insufficiency: a proof-of-concept study using copeptin-derived ratios
Source: J Endocrinol Invest. 2026 Apr 24;49(8):1889–901. doi: 10.1007/s40618-026-02874-8 (PMC13486049; doi:10.1007/s40618-026-02874-8)
Supplement: Supplementary file 1 [file 40618_2026_2874_MOESM1_ESM.docx]

**Table 1** Salivary cortisol (F), cortisone (E) and cortisol/cortisone ratio (F/E) median values in healthy subjects and in PAI patients

|  | Healthy subjects (n=43) | PAI patients (n=19) | |
| --- | --- | --- | --- |
|  |  | **T0** | **T1** |
| F0 (ng/ml) | 8.20 [5.30-11.60] | 0.47 [0.47-2.25] | 0.47 [0.47-1.50] |
| F1(ng/ml) | 8.20 [4.50-15.00] | 29.44 [12.60-102.4] | 24.95 [19.24-74.21] |
| F2 (ng/ml) | 4.30 [2.30-7.30] | 7.67 [1.39-16.63] | 4.50 [1.97-8.90] |
| F3 (ng/ml) | 2.80 [1.70-4.40] | 3.10 [0.64-4.88] | 5.91 [1.43-22.04] |
| F4 (ng/ml) | 1.70 [1.00-3.00] | 1.13 [0.47-2.26] | 2.26 [0.47-6.95] |
| F5 (ng/ml) | 0.90 [0.60-1.20] | 0.47 [0.47-1.02] | 1.10 [0.47-2.48] |
| E0 (ng/ml) | 25.00 [19.20-29.00] | 2.00 [2.00-3.70] | 2.00 [2.00-3.23] |
| E1 (ng/ml) | 29.30 [19.40-36.00] | 42.70 [33.77-60.22] | 43.99 [36.88-49.54] |
| E2 (ng/ml) | 18.30 [14.70-26.30] | 20.23 [9.91-30.60] | 21.96 [10.93-31.65] |
| E3 (ng/ml) | 13.00 [11.10-17.80] | 10.50 [4.48-20.18] | 10.75 [7.04-18.72] |
| E4 (ng/ml) | 11.00 [6.80-16.20] | 6.59 [2.64-12.99] | 11.46 [3.04-21.18] |
| E5 (ng/ml) | 5.40 [4.10-8.70] | 2.29 [2.00-7.50] | 4.31 [2.00-9.14] |
| F/E0 | 0.34 [0.27-0.41] | 0.24 [0.24-0.71] | 0.24 [0.24-0.29] |
| F/E1 | 0.29 [0.21-0.41] | 0.51 [0.34-1.77] | 0.57 [0.41-1.50] |
| F/E2 | 0.23 [0.17-0.33] | 0.28 [0.13-0.64] | 0.22 [0.18-0.42] |
| F/E3 | 0.19 [0.15-0.26] | 0.21 [0.16-0.30] | 0.27 [0.16-1.65] |
| F/E4 | 0.18 [0.13-0.21] | 0.22 [0.17-0.24] | 0.22 [0.15-0.30] |
| F/E5 | 0.17 [0.13-0.21] | 0.24 [0.18-0.24] | 0.24 [0.21-0.25] |

Data are reported as median [25^th^-75^th^ percentile]

Abbreviations: PAI, primary adrenal insufficiency; F, cortisol; E, cortisone

**Fig 1** Cortisol (F) and Cortisone (E) profile in PAI patients at T0

**Fig 2** Cortisol (F) and Cortisone (E) profile in PAI patients at T1
